# Local and regional factors influence the structure of treehole metacommunities

**DOI:** 10.1186/1472-6785-8-22

**Published:** 2008-12-19

**Authors:** Christopher J Paradise, Jarrod D Blue, John Q Burkhart, Justin Goldberg, Lauren Harshaw, Katherine D Hawkins, Benjamin Kegan, Tyler Krentz, Leslie Smith, Shawn Villalpando

**Affiliations:** 1 Biology Department, Davidson College, Davidson, NC, USA; 2 Geology and Environmental Studies Departments, Middlebury College, Middlebury, VT, USA; 3 Classics Department, Trinity University, San Antonio, TX, USA; 4 Biology Department, Appalachian State University, Boone, NC, USA

## Abstract

**Background:**

Abiotic and biotic factors in a local habitat may strongly impact the community residing within, but spatially structured metacommunities are also influenced by regional factors such as immigration and colonization. We used three years of monthly treehole census data to evaluate the relative influence of local and regional factors on our study system.

**Results:**

Every species responded to at least one of three local environmental factors measured: water volume, leaf litter mass, and presence of a top predator. Several species were affected by water volume, and a non-exclusive group of species were influenced by leaf litter mass. Relative abundance of *Aedes triseriatus *was higher in treeholes with higher volumes of water, and relative abundances of three out of six other species were lower in treeholes with higher volumes of water. Leaf litter mass positively affected densities of *Aedes triseriatus *and relative abundance of several dipteran species. The density of the top predator, *Toxorhynchites rutilus*, affected the relative abundance of the two most common species, *A. triseriatus *and *Culicoides guttipennis*. Treeholes with *T. rutilus *had an average of two more species than treeholes without *T. rutilus*. We found little evidence of synchrony between pairs of treeholes, either spatially or temporally. There were high levels of spatial and temporal turnover, and spatial turnover increased with distance between patches.

**Conclusion:**

The strong effects of water volume, leaf litter mass, and presence of a top predator, along with the high temporal turnover strongly suggest that species presence and density are determined by local factors and changes in those factors over time. Both low water volume and high predator densities can eliminate populations in local patches, and those populations can recolonize patches when rain refills or predators exit treeholes. Population densities of the same species were not matched between pairs of treeholes, suggesting variation in local factors and limited dispersal. Distance effects on spatial turnover also support limitations to dispersal in the metacommunity, and we conclude that the weight of evidence favors a strong influence of local factors relative to regional factors.

## Background

Consideration of both local and regional processes is essential in studies of communities found in discrete and spatially separated habitat patches [1-3], such as phytotelmata, ponds, lakes, decomposing logs, rock pools, and fragmented habitats. Dispersal by species among habitats may affect dynamics and community structure in local habitats [1,4]. Metacommunity ecology is the study of the factors that influence the dynamics in such spatially-structured communities. Populations in these habitats may persist for a time or go locally extinct, after which they be rescued from extinction by colonization events [1]. Exploration of such metacommunity dynamics may increase understanding of community structure at both the local and regional scales [2].

Several related paradigms have been developed to understand metacommunity dynamics, and all make predictions about the relative influence of local and regional factors. Predictions of the different paradigms are not mutually exclusive, and several recent studies of various systems have found support for more than one paradigm in describing the dynamics of metacommunities [3-7]. Metacommunities have been shown to be affected both by abiotic and biotic conditions within a local habitat, as well as regional factors, such as distance between patches and dispersal ability of species.

Local factors may have a relatively larger influence on community composition than regional factors, as in the species sorting paradigm. Here, variation among patches in local conditions, such as resource availability and predation, causes differences in local demography, the outcome of interactions, and community composition [3,5-7]. Different species perform better in some patch types than others [1,4], and dispersal among patches is not so frequent that species regularly occur in sink patches [3]. Although regional factors have less influence than local factors, low dispersal rates allow changes in local conditions to be tracked by species, resulting in temporal changes in species composition [1].

As dispersal among local communities increases or distance between them decreases, different dynamics in the metacommunity are expected. With a moderate amount of dispersal or distance between patches, resident populations undergo repeated extinctions and colonization, local species diversity increases, and communities close together tend towards a high degree of similarity [4]. Species relative abundances will shift locally over time, but these shifts will not correlate to any temporal changes in environmental conditions [4]. Populations may be eliminated due to their presence in a sink habitat, presence of predators, or presence of superior competitors. But as dispersal increases or distance between habitats decreases further, the mass effects paradigm predicts existence of populations of species in habitats where they could not exist otherwise [1-3]. Here, regional factors are stronger relative to local factors, such that migration can rescue populations in suboptimal habitat. Community similarity between patches will be high and will also correlate to the distance between patches and depend upon the dispersal abilities of resident species [4].

### Treeholes: local and regional characteristics

Macroinvertebrate communities in treeholes of central North Carolina consist mainly of Diptera and Coleoptera. The most common insects in NC treeholes are the culicid *Aedes triseriatus *(Say) and the ceratopogonid *Culicoides guttipennis *(Coquillet) [8,9]. Other dipterans include the culicids *A. albopictus *(Skuse), *Orthopodomyia signifera *(Coquillet), *Toxorhynchites rutilus *(Coquillet), and *A. hendersoni *Cockerell, the syrphid *Mallota posticata *(Fabr.), the psychodid *Telmatoscopus albipunctatus *(Williston), and a dolichopodid (*Systenus *sp.) [8]. The lone coleopteran we have found is the scirtid beetle *Helodes pulchella *(Guerin) [9].

High levels of the resources of water and leaf litter positively influence individuals, populations, and the entire insect community [10-16]. Most treehole insects feed on microorganisms, which break down leaf litter, but they also survive on dissolved organic carbon, sediment, and any other organic material within treeholes [17]. Several biotic interactions have strong effects on treehole populations, including predator-prey interactions [18-21], and competition [22,23].

Treeholes are discrete, spatially separated communities, with obligate insect inhabitants that live within them for part of their life cycle and then emerge as adults and disperse to new habitats. Larval populations can be ephemeral, and some species may be present during only a limited time of the year, but several species are present throughout the growing season. Seasonal activity patterns and oviposition site selection may promote species diversity regionally and reduce diversity locally, as biotic interactions change depending on the inhabitants. As a result of their complex life cycles, the dynamics and diversity of local insect communities could also be influenced by regional factors of dispersal and migration. In addition, treeholes are variable in space and time and are subject to drought, a major disturbance to these communities that may be a primary factor contributing to temporal changes in community structure [24-26].

### Predictions for treehole metacommunities

The degree to which treehole insect metacommunities are influenced by local relative to regional factors is not well known. Ellis et al. [3] examined over two decades worth of data on just the mosquito species inhabiting Florida treeholes and concluded that these habitats exhibit aspects of species sorting and patch dynamics. They found strong influences of environmental conditions, high spatial and temporal turnover, and asynchronous dynamics, which we predict will be true for the entire insect community. Treeholes show significant interpatch heterogeneity [10,11,16], leading to our prediction that local conditions would be most influential in determining treehole community dynamics.

Specifically, we predict that we will observe strong influences of water volume, leaf litter mass and predation on relative abundance and presence of individual species, as has been shown in other studies [10,27,28]. Mosquitoes will be strongly affected by water volume, whereas several species that have been observed in treeholes with little standing water will be less affected by water volume. We predict that all species are potential prey for the top predator, *T. rutilus*, and their densities will be lower in treeholes with the predator present.

We also predict patch occupancy will vary with environmental conditions such as water and leaf litter availability and presence of predators. During times when treeholes are filled with water and leaf litter, occupancy of each species will be high. Conversely, when treeholes begin to dry and leaf litter decomposes during the late summer, patch occupancy and colonization should be lower. This should lead to a temporal effect, with high temporal turnover over the course of the season. The species found in the highest densities within individual treeholes, *A. triseriatus *and *C. guttipennis*, will also be the most widespread, having the highest colonization and occupancy proportions within the metacommunity [8,16]. Yet even for these species, populations in different treeholes will not be synchronized due to the random nature of dispersal [29] and the influence of local environmental factors.

We further predict that as distance between patches increases, asynchrony and spatial turnover (i.e., community dissimilarity) will increase due to limitations of dispersal ability and the stochastic nature of treehole discovery [29]. The large variation of environmental conditions among treeholes and rapid temporal changes within treeholes will lead to high spatial and temporal turnover. These predictions were tested using data from several natural, unmanipulated metacommunities, sets of treeholes within a stand of forest, sampled repeatedly for three years. Our study provides strong support for the primacy of environmental factors in determining local communities within a metacommunity, but also shows slight effects of dispersal and distance between patches.

## Methods

We tested our predictions by comparing relative abundance, presence, patch occupancy, colonization, spatial synchrony, and turnover to both local and regional factors. Local factors were water volume, leaf litter mass and top predator density. The regional factor was distance between patches (treeholes). We used analyses similar to Ellis et al. [3], but our data set included treeholes with conditions that these authors excluded, because several non-mosquito species are likely to be found in these conditions. For instance, the midge *C. guttipennis *can be found in water-filled treeholes, but also in treeholes with little standing water.

### Study sites

The study was performed in three sites over three consecutive field seasons, beginning in May 2004 and ending September 2006. Two sites were located on the Davidson College Ecological Preserve (DCEP, Davidson, NC; Site 1 centered on 35° 30' 37" N, 80° 49' 48" W and site 2 centered on 35° 30' 14" N, 80° 50' 02" W), and the third site was located on the Davidson College Lake Campus (DCLC, Mt. Mourne, NC, centered on 35° 31' 48" N, 80° 52' 58" W). DCEP sites were located almost 1 km apart, and were separated by at least one power line right-of-way. The DCLC is > 5 km from the two DCEP sites, with roads, a reservoir, suburban developments, and fragmented habitats in between. The forests within the sites were similar, in that they were second growth, mixed deciduous forests, consisting mainly of dogwood (*Cornus florida *L.), oaks (*Quercus *spp.), tulip poplar (*Liriodendron tulipifera *L.), maples (*Acer *spp.), and other deciduous trees.

### Censuses

At each site, we initially selected between 5 and 10 treeholes to monitor. Treeholes among sites differed in size, volume of water and amount of leaf litter. Total volume of individual treeholes was estimated to range from 200 to over 7,500 mL, with water volumes ranging from 0 to 7,000 mL, and wet mass of leaf litter ranged from 0 to 250 g. Twenty treeholes were sampled in 2004; four of those were dropped at the end of 2004 because they no longer held water. Thirteen treeholes were added at the beginning of 2005. All 29 treeholes were sampled for the next two seasons, except for four that were dropped in August 2006 because they had dried up. Fifteen treeholes were censused over the three full field seasons, a field season being the period of time when most species were active as larvae, from March or April to October or November.

Each month we removed all water by suction from each treehole, quantified volume, and poured extracted water into pans [16]. We extracted coarse particulate organic matter (hereafter, leaf litter), placed it into a tared beaker and determined its wet mass using a PocketPro digital balance (Acculab, Edgewood, NY). We collected a subsample of sediment (~5–100 mL, depending upon size of treehole) to estimate larval insect densities within the sediment. For each subsample, we homogenized the sediment with a ladle or small spoon, withdrew a sample, estimated its volume, and placed it in an enamel pan. We added a small amount of distilled water to disperse the sediment. We counted insect larvae by species and instar or size class (i.e., when instars could not be determined reliably, such as for scirtid beetles) in each component. We carefully replaced all sediment, litter, and water, in that order, after each census.

We recorded spatial UTM (Universal Transverse Mercator) coordinates using a Garmin GPS III Plus unit (Garmin International, Olathe, KS). Coordinates were entered into a database, and a spatial map of each site was generated. We also used an online UTM-finder  to compare groundtruthed UTMs of landmarks to those online and visible in aerial photographs. This allowed us to determine the reliability of GPS coordinates obtained in a forest setting. Using simple geometry, we determined the distance between treehole pairs. We groundtruthed a subset of these estimates using an infrared rangefinder (Bushnell Yardage Pro Sport 450, Overland Park, KS) to validate distances.

### Analyses of environmental variation

We examined relative abundance of individual species compared to water volume and leaf litter mass using randomization regressions [30]. Relative abundance is the proportion of all larvae in a treehole in one census belonging to a particular species. For each species, we used only treeholes that had non-zero densities of that species, as we were interested in the relationship between water or leaf litter and the relative abundance of the insect, when present. We linearized relationships between relative abundance and volume or mass by taking log transforming water volume (in liters) and the inverse of relative abundance. Randomization tests were performed using RT: A Program for Randomization Testing (v. 2.1) [30], with significance and 95% confidence intervals calculated using 10,000 randomizations [30]. We performed these tests for the seven species for which we had sufficient data: *A. triseriatus*, *T. rutilus*, *C. guttipennis*, *H. pulchella*, *M. posticata*, *T. albipunctatus*, and *Systenus *sp.

We used logistic regression to test for effects of water volume and leaf litter wet mass on the presence-absence of the seven species mentioned above plus the mosquito *O. signifera*. As pointed out in Ellis et al. [3], non-independence of data leads us to regard logistic regression as an approximate test. Some species exhibited distinct seasonality. *Toxorhynchites rutilus *larvae were not observed in March or April and *M. posticata *larvae were observed only in May, June, and July. *Systenus *sp. larvae were never observed in September or October. For each of these species, data from months when each was not observed were eliminated.

We tested for a relationship between the two most common species, *A. triseriatus *and *C. guttipennis*, and the predatory *T. rutilus*. We performed randomization regressions on log-transformed densities of prey and predator for any treehole in which we found the prey, the predator, or both. In addition, a randomization two-sample test was performed on species richness in treeholes where *T. rutilus *was present vs. treeholes where it was absent.

### Patch occupancy and colonization analyses

Occupancy is the proportion of treeholes occupied by a species during each census [3]. Bootstrap means with 1,000 resamplings were calculated to examine mean proportion occupancy over time. We compared the monthly proportion occupancy to both the monthly average log-transformed water volume and monthly average log-transformed leaf litter wet mass using randomization regressions as described above. We determined proportion occupancy for six species for which we had sufficient data. Treeholes were eliminated from the analysis of particular species if they never contained that species. For analysis of *T. rutilus *and *M. posticata*, we did not use data from months when those species did not occur, as noted above.

For colonization, we counted larvae of several species by instar or size class and we used those data to record colonization events (or hatchings after water refilled treeholes) based on appearance of first instar or small larvae. We did this for *C. guttipennis*, *A. triseriatus*, *H. pulchella*, and *T. rutilus*. For *A. triseriatus*, we only considered the proportion of treeholes that actually contained water, as eggs may have been laid in a dry treehole, but they would not hatch until water returns. For *M. posticata *and *T. albipunctatus*, we defined a colonization event as the appearance of a species after a treehole went from having no larvae of that species to containing larvae of that species. This may miss some colonization events if they occur while the treehole is already occupied by that species, so it is a conservative measure.

### Spatial synchrony analysis

We used lag-zero cross correlations of log-transformed densities using Minitab (v. 13.1) to examine fluctuations in species' densities across pairs of treeholes. Values close to or below zero would mean asynchrony between pairs, and would indicate low or localized dispersal. All data were used, unless both treeholes in a pair at a particular point in time had densities of zero. Treeholes that never contained a particular species were eliminated for analysis of that species.

We calculated bootstrap means for each species, which allows for non-independence of treehole pairs and repeated samplings [3]. Randomized linear regressions (10,000 randomizations performed on RT v2.1 were used to examine the relationship between mean synchrony for a pair of treeholes vs. distance between those treeholes. It is unlikely that dispersal occurs between treeholes separated by great distances, so we performed regressions for each species using treehole pairs within sites only. The species tested were *A. triseriatus*, *T. rutilus*, *C. guttipennis*, *H. pulchella*, *M. posticata*, and *T. albipunctatus*.

### Species composition and turnover analyses

Spatial turnover, a measure of community dissimilarity, was calculated as [(A_obs _+ B_obs_)/(S_A _+ S_B_)] × 100, where A_obs _is the number of species found in site A but not in site B, B_obs _is the number of species found in site B but not in Site A, S_A _is the species richness in site A, and S_B _is the species richness in site B [3]. This was calculated for all treehole pairs within a site that contained at least one species in one of the two treeholes being compared. We computed bootstrap means of all within-site pairs (1,000 resamplings with 95% CI).

Temporal turnover was calculated for each treehole by comparing data from one month to the next, for as many values as there were consecutive pairs of data through time for a treehole. It was calculated as [(X_obs _+ Y_obs_)/(S_X _+ S_Y_)] × 100, where X_obs _is the number of species found in a treehole in month X but not in month Y, Y_obs _is the number of species found in a treehole in month Y but not in month X, S_X _is the species richness in month X, and S_Y _is the species richness in month Y [31]. The comparison of the last month of one field season and the first month of the next was excluded because of the long interval of time between these months. We averaged temporal turnover for each time sequence (e.g., March to April, 2005) across all treeholes for which we had data from those two months. We also computed bootstrap means (1,000 resamplings with 95% CI) for each treehole.

Randomization regressions with 10,000 randomizations of mean spatial turnover over all three field seasons vs. distance between pairs of treeholes were performed using RT v2.1 as described. Randomization regressions with 10,000 randomizations were performed on mean temporal turnover of a treehole vs. mean water volume for that treehole, using RT v2.1.

## Results

### Environmental variation

**Figure 1:**
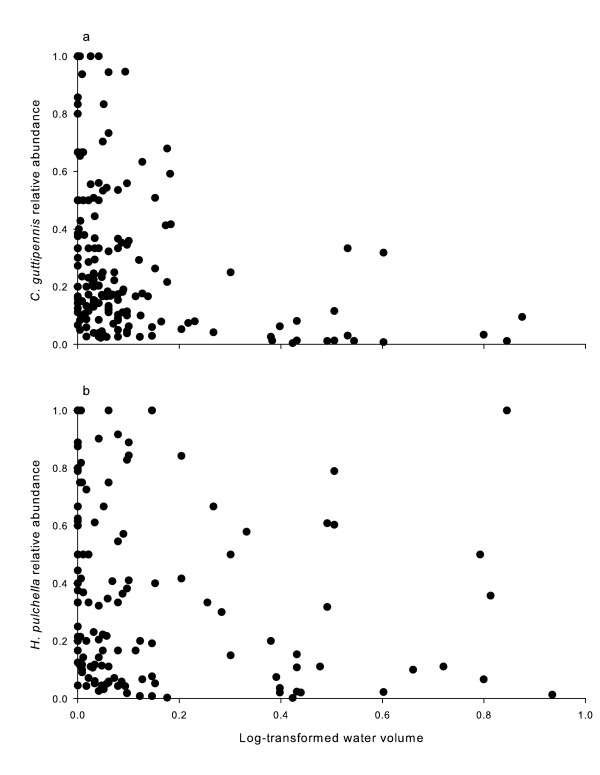
**Insect relative abundance *vs*. log-transformed water volume**. Relationship between *C. guttipennis *(a) and *H. pulchella *(b) relative abundance and log-transformed water volume. Each point represents one treehole census.

**Figure 2:**
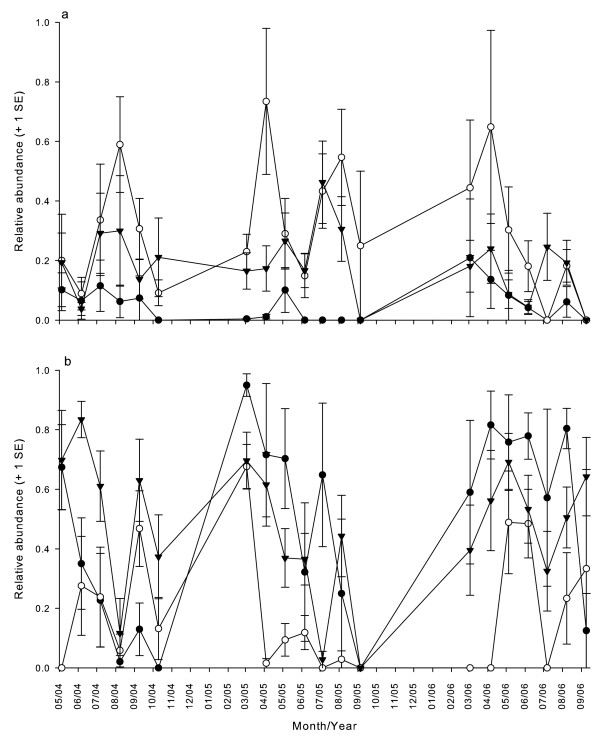
**Relative abundances over time, within sites, of the two most common insects**. a. *Culicoides guttipennis *relative abundances. b. *Aedes triseriatus *relative abundances. Relative abundance is the proportion of all larvae in a treehole in one census belonging to a particular species. Closed circles are for site 1 on the Ecological Preserve (DCEP), open circles are for site 2 (DCEP), and triangles are for site 3 on the Lake Campus (DCLC). Error bars represent 1 SE.

*Aedes triseriatus *relative abundance increased with increasing water volume, *C. guttipennis *and *Systenus *sp. relative abundances decreased with increasing water volume and leaf litter mass, and relative abundance of *H. pulchella *decreased with increasing water volume only (Fig 1; Table 1). Relative abundance of *T. albipunctatus *decreased with increasing leaf litter mass (Table 1). Relative abundances for the most common species, *A. triseriatus *and *C. guttipennis*, varied across sites (23% and 34% of variation was attributable to differences among the three sites, respectively), and within site they varied temporally (Fig 2). The temporal variation component of these data was 42.1% for *A. triseriatus *and 32.5% for *C. guttipennis*.

**Table 1:** Effects of log-transformed water volume and leaf litter mass on relative abundances of treehole insects

Species	*N*	*R*^2^	*F*	*P*_*F*_	Parameter	Coefficient	SE	*t*	*P*_*t*_
*A. triseriatus*	233	0.041	6.00	0.003	Water vol	0.22	0.10	2.19	0.03
					Leaf mass	0.042	0.03	1.33	0.18

*T. rutilus*	89	0.028	2.27	0.11	Water vol	-0.51	0.32	-1.57	0.12
					Leaf mass	0.21	0.11	1.97	0.052

*C. guttipennis*	202	0.32	48.52	0.0001	Water vol	1.35	0.21	6.30	<0.001
					Leaf mass	0.22	0.06	3.98	<0.001

*H. pulchella*	160	0.088	8.70	0.004	Water vol	0.63	0.26	2.44	0.016
					Leaf mass	0.11	0.073	1.52	0.13

*M. posticata*	45	0.14	4.43	0.019	Water vol	0.54	0.36	1.51	0.14
					Leaf mass	0.22	0.11	1.92	0.06

*T. albipunctatus*	98	0.078	5.09	0.008	Water vol	0.25	0.34	0.74	0.46
					Leaf mass	0.29	0.10	2.84	0.005

*Systenus *sp.	21	0.51	11.39	0.007	Water vol	1.22	0.45	2.69	0.015
					Leaf mass	0.44	0.15	2.97	0.01

Results of randomization regressions on inverse-transformed relative abundances vs. the log-transformed water volume and leaf litter mass. *N *= sample size, *R*^2 ^= proportion of variation explained by the model, *F *= regression model test statistic, *P*_*F *_= probability value associated with *F *statistic, SE = standard error of the coefficient estimate, *t *= test statistic for coefficient, *P*_*t *_= probability associated with t-statistic. Y-intercepts not shown, but all were significantly greater than zero (*P *< 0.001). *Aedes triseriatus *relative abundances were not transformed, as transformation did not improve linearity.

The amount of water or leaf litter present affected the presence of five species (Table 2). The logistic model using log-transformed water volume and leaf litter mass fit well for all species but the two most common species, *A. triseriatus *and *C. guttipennis *(Table 2). Despite that, both species were positively affected by water volume. For *T. rutilus*, water volume had a significant positive effect and leaf litter mass had a significant negative effect on the probability of larval presence. The probability of *M. posticata *presence was higher in treeholes with higher water volumes, and the probability of *H. pulchella *presence was higher in treeholes with higher leaf litter masses (Table 2).

**Table 2:** Effects of log-transformed water volume and leaf litter mass on presence-absence of treehole insects

Species	*χ*^2 ^*GOF*	*P*_*χ*_	Parameter	Coefficient	SE	*z*	*P*_*z*_
*A. triseriatus*	382.56	<0.001	Water vol	1.37	0.11	11.92	<0.001
			Leaf mass	0.09	0.25	0.37	0.71

*O. signifera*	124.62	1.000	Water vol	0.90	0.54	1.69	0.09
			Leaf mass	0.66	0.56	1.19	0.23

*T. rutilus*	205.38	0.29	Water vol	1.36	0.16	8.56	<0.001
			Leaf mass	-0.69	0.22	-3.22	0.001

*C. guttipennis*	241.89	0.013	Water vol	0.34	0.08	4.23	<0.001
			Leaf mass	-0.13	0.18	-0.74	0.46

*H. pulchella*	202.51	0.34	Water vol	0.14	0.08	1.68	0.09
			Leaf mass	0.63	0.18	3.51	<0.001

*M. posticata*	214.39	0.16	Water vol	0.36	0.13	2.80	0.005
			Leaf mass	0.18	0.24	0.77	0.442

*T. albipunctatus*	210.24	0.22	Water vol	0.15	0.10	1.56	0.12
			Leaf mass	0.054	0.21	0.26	0.79

*Systenus *sp.	205.88	0.28	Water vol	-0.07	0.21	-0.34	0.74
			Leaf mass	0.92	0.36	2.56	0.01

Logistic regression results on presence-absence. *N *= 514 for each species. *χ*^2 ^*GOF *= chi-square goodness of fit. *P*_*χ *_= probability value associated with *χ*^2 ^statistic, SE = standard error of the coefficient estimate, *z *= test statistic for coefficient, *P*_z _= probability associated with z-statistic. Y-intercepts not shown, but all were significantly less than zero (*P *< 0.001).

**Figure 3:**
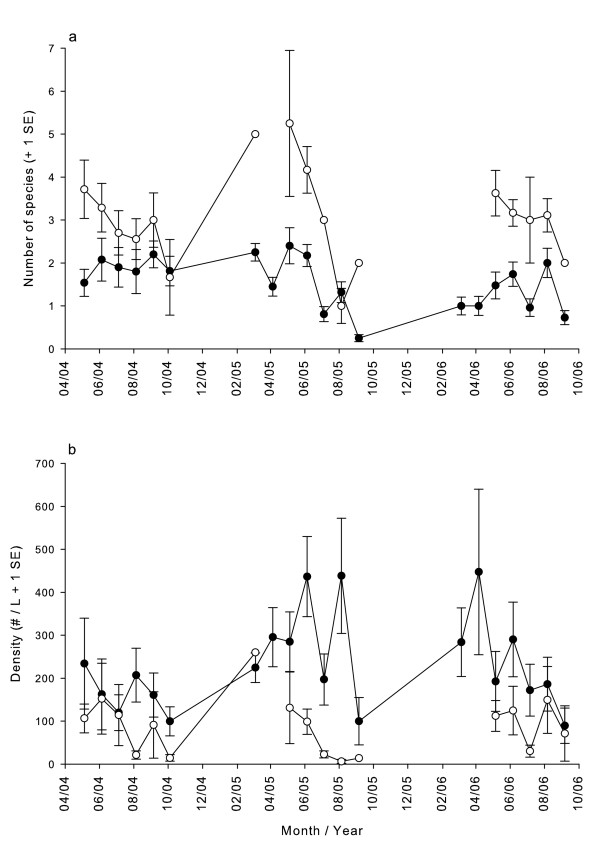
**Richness and insect density over time in treeholes with and without predation**. a. Species richness over the three year study period in treeholes with and without *T. rutilus*. b. Total insect (not including *T. rutilus*) density over the three year study period in treeholes with and without *T. rutilus*. Means are based on variable sample sizes, depending on how many of the 29 treeholes had *T. rutilus *and how many did not. If only early instar (instars I and II) *T. rutilus *were found, the predator was not counted as being present for the purposes of calculating these means. Closed circles are means without *T. rutilus *present, and open circles are means with *T. rutilus *present. Error bars represent 1 SE.

Species richness was higher and total insect density was lower in the presence of *T. rutilus *(Fig 3). Bootstrapped mean species richness over three field seasons in the presence of *T. rutilus *was 3.28 (± 0.17 SE) and in its absence was 1.42 (± 0.06 SE). A two-sample randomization test indicated that these two means were significantly different (*df *= 512, *P *= 0.0001). Randomized regressions revealed that densities of both *A. triseriatus *and *C. guttipennis *declined significantly with increasing *T. rutilus *densities. Density of the predator explained 8.8% of the variation in *A. triseriatus *densities (log *A. triseriatus *density = 1.73 - 0.62·(log *T. rutilus *density); *F*_1,256 _= 24.64, *P *= 0.0001) and 28.2% of the variation in *C. guttipennis *densities (log *C. guttipennis *density = 1.61 - 1.19·(log *T. rutilus *density); *F*_1,253 _= 99.14, *P *= 0.0001).

### Patch occupancy and colonization

**Figure 4:**
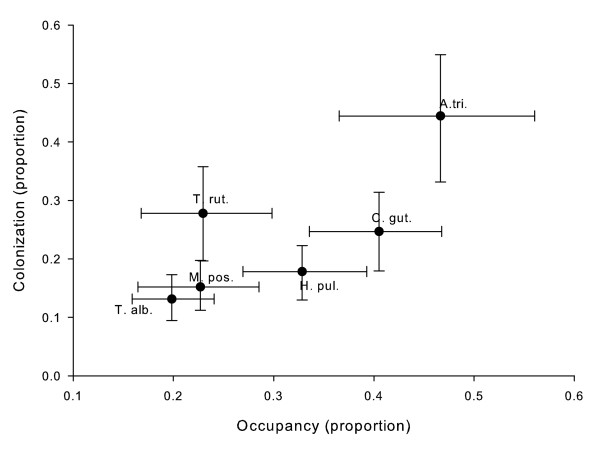
**Colonization *vs*. occupancy for treehole species**. Proportion colonization, the proportion of treeholes in any one census that had been colonized between the census and the previous one, vs. proportion occupancy, the proportion of treeholes occupied by a species during a census, for each of six treehole species. Means ± 1 SE are plotted. See text for methods of estimation of colonization and occupancy, as they varied among species.

*Aedes triseriatus *and *C. guttipennis *had the highest occupancy (Fig 4). *Aedes triseriatus *occupancy was positively related to average water volume (Table 3), suggesting that occupancy is higher in larger treeholes with more stable hydroperiods, a finding inconsistent with Ellis et al. [3]. *Aedes triseriatus *also had the highest colonization proportion, as measured by the proportion of treeholes with standing water that contained first instar mosquitoes during any monthly census. Proportion colonization of *A. triseriatus *was not significantly related to either water volume or leaf litter mass (*P *> 0.05).

**Table 3:** Effects of log-transformed water volume and leaf litter mass on proportion occupancy

Species	*N*	*R*^2^	*F*	*P*_*F*_	Parameter	Coefficient	SE	*t*	*P*_*t*_
*A. triseriatus*	20	0.58	14.32	0.0003	Y-intercept	0.063	0.08	0.77	0.45
					Water vol	0.27	0.06	4.26	0.001
					Leaf mass	0.084	0.19	0.44	0.67

*T. rutilus*	17	0.37	5.80	0.015	Y-intercept	0.012	0.08	0.15	0.88
					Water vol	0.20	0.06	3.40	0.007
					Leaf mass	-0.32	0.16	-2.03	0.062

*C. guttipennis*	20	0.25	4.12	0.033	Y-intercept	0.19	0.08	2.41	0.017
					Water vol	0.12	0.06	1.87	0.012
					Leaf mass	0.16	0.19	0.82	0.42

*H. pulchella*	20	0.23	3.77	0.044	Y-intercept	0.16	0.07	2.28	0.035
					Water vol	0.15	0.06	2.66	0.017
					Leaf mass	-0.14	0.17	-0.84	0.42

*M. posticata*	8	0.16	1.69	0.28	Y-intercept	0.044	0.11	0.42	0.69
					Water vol	0.12	0.08	1.63	0.16
					Leaf mass	-0.02	0.22	-0.11	0.92

*T. albipunctatus*	20		1.93	0.18	Y-intercept	0.11	0.05	2.12	0.049
					Water vol	0.048	0.04	1.23	0.24
					Leaf mass	0.075	0.12	0.64	0.54

Results of randomization regressions of proportion occupancy vs. log-transformed water volume and leaf litter mass. *N *= number of months used. Number varies because some species are not active year round. *R*^2 ^= proportion of variation explained by the model, *F *= regression model test statistic, *P*_*F *_= probability associated with *F *statistic, s.e. = standard error of the coefficient estimate, *t *= test statistic for coefficient, *P*_*t *_= probability associated with t-statistic.

*Culicoides guttipennis *and *H. pulchella *had similar proportions of occupancy and colonization (Fig 4). As with *A. triseriatus*, both species showed higher occupancy in treeholes with larger water volumes (Table 3). These two species had Y-intercepts significantly different from zero, suggesting high occupancy even when treeholes contained little or no water. All other species had occupancies not significantly different from zero when water volume was zero (Table 3). *Toxorhynchites rutilus *occupied a lower proportion of treeholes than *C. guttipennis*, but also showed higher occupancy in treeholes with larger water volumes. No randomization regressions on proportion colonization for these three species were significant (*P *= 0.05, 0.39, and 0.17 for *C. guttipennis*, *H. pulchella*, and *T. rutilus*, respectively).

*Mallota posticata *and *T. albipunctatus *each had ~20% occupancy and ~15% colonization. Both *M. posticata *and *T. albipunctatus *may be locally abundant, but are present in only a low proportion of treeholes (Fig 4).

### Spatial synchrony

**Figure 5:**
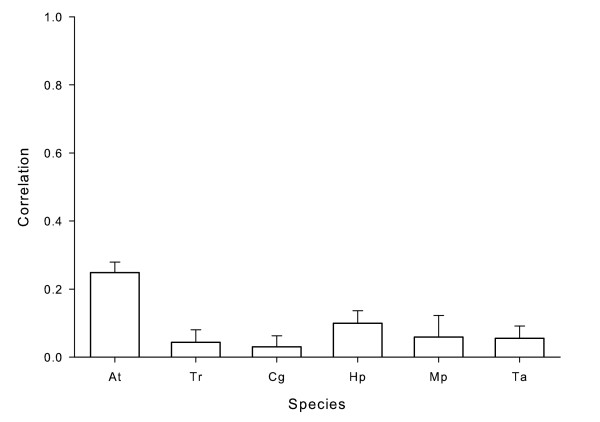
**Spatial synchrony for six treehole species**. Bootstrap mean correlations (± 95% CI) of log-transformed densities of individual species among pairs of treeholes within each site, pooled across all sites. Values close to 1 indicate synchronous populations, while values close to 0 are reflective of asynchronous populations.

The mean pooled within-site cross correlations for density of each species were significantly different from 1, indicating asynchrony (*P *< 0.001 for each species; Fig 5). Density correlations were not significantly different from 0 for *T. rutilus*, *C. guttipennis*, and *M. posticata *(*P *= 0.46, 0.06, and 0.09, respectively), indicating asynchrony; all other species were significantly different from 0 (*P *< 0.001). Our spatial synchrony estimates for mosquitoes (*A. triseriatus *and *T. rutilus*) were qualitatively similar to those of Ellis et al. [3]. Further, the patterns in presence-absence synchrony were similar to correlations for density (data not shown).

Treeholes within a site ranged from 8 to 404 m apart (median = 161 m). We found synchrony to be independent of distance for that range; regressions of density correlations against distance were not significant for any species (*A. triseriatus*: *F*_1,151 _= 1.99, *P *= 0.16; *T. rutilus*: *F*_1,81 _= 0.94, *P *= 0.34; *C. guttipennis*: *F*_1,167 _= 2.65, *P *= 0.11; *H. pulchella*: *F*_1,132 _= 0.27, *P *= 0.60; *M. posticata*: *F*_1,63 _= 0.01, *P *= 0.90; and *T. albipunctatus*: *F*_1,123 _= 0.79, *P *= 0.38).

### Species composition and turnover

**Figure 6:**
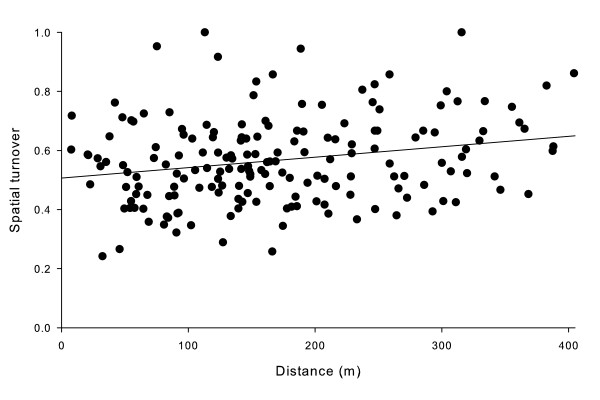
**Spatial turnover for pairs of treeholes**. Spatial turnover for each pair of treeholes within sites is averaged across all months in which both treeholes were sampled. The best-fit line is shown, with coefficients from text.

The overall bootstrap mean spatial turnover was 0.73 (± 0.01 SE) for all within-site comparisons. There was a significant positive relationship between spatial turnover and distance between treehole pairs for all within-site comparisons (Fig 6; spatial turnover = 0.51 + (0.00035·distance (m)); *F*_1,176 _= 9.45; *P *= 0.002). In addition, the Y-intercept was significantly greater than 0 (*t *= 22.90, *P *< 0.001), indicating that spatial turnover was high even for treeholes next to each other.

**Figure 7:**
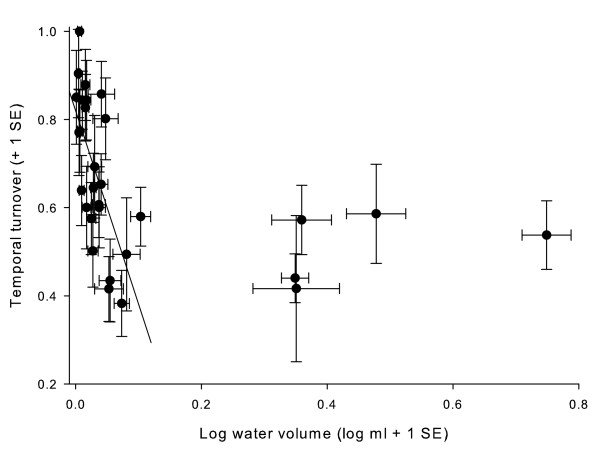
**Temporal turnover *vs*. water volume**. Average temporal turnover, or change in community composition from one census to the next, if the two were one month apart, for each treehole vs. log-transformed water volume. Sample size ranged from 6 to 16, depending on duration of sampling. The best fit line shown is for the subset of treeholes with mean water volumes < 300 mL.

Temporal turnover ranged from 0.38 to 1.00. The data for the regression with log water volume were non-normal and no transformation could be found to reduce heteroscedasticity. However, the subset of treeholes that had long-term mean water volumes of less than 300 mL had a negative linear relationship with log water volume (randomization regression: temporal turnover = 0.82 - 4.39·(log water volume); *F*_1,23 _= 16.18, *P *= 0.001), with temporal turnovers that ranged from 0.38 to 1.00 (Fig 7). Turnover ranged from 0.40 to 0.57 in a group of five large treeholes that contained more than 1,000 ml of water (Fig 7). Temporal turnover was thus highest in small treeholes with lower average water volumes.

## Discussion

### Environmental effects

Variation in environmental factors explains much of the variation among local treehole populations and communities [12,13,15,16,19,32,33]. Our treeholes varied in water volume, leaf litter mass, and predation intensity, over time and among patches. Litter abundance is positively correlated with richness, total biomass, and densities of individual species [10,16,34,35]. We found leaf litter to exert negative effects on relative abundance of *C. guttipennis*, *T. albipunctatus*, and *Systenus *sp. This is somewhat surprising for *C. guttipennis *as populations of this species respond positively to high levels of leaf litter [36].

As predicted, *A. triseriatus *was more likely to be found in higher relative abundances, and *C. guttipennis*, *H. pulchella*, and *Systenus *sp. were more likely to be found in lower relative abundances, in treeholes with more water. Larger treeholes with larger openings are easier to find by dispersing females, hold more water, and are less susceptible to drying out than smaller treeholes [11,24]. While *C. guttipennis *and *H. pulchella *may be relatively drought-tolerant (Table 3) [16], mosquitoes are less so [24]. Mosquito eggs hatch quickly after treeholes fill from rain [3,37], however, if treeholes remain dry, eggs will not hatch, and any mosquito larvae will die, which corresponds to our finding that mosquito presence-absence was highly dependent upon water volume. Thus, treeholes with more water are dominated by *A. triseriatus*, while *C. guttipennis *and *H. pulchella *dominate when water volumes are low, which conforms to patterns from Paradise [16].

Locally, variability in water volume may affect community structure by either eliminating populations or allowing persistence [29]. Drought is a large disturbance to these communities, and water volumes may fluctuate dramatically during the summer [14,24-26]. We found high temporal turnover in individual treeholes, a finding consistent with other studies from these and other container communities [3,7,29,32,37]. Because local factors tend to rapidly change in treeholes [14,16], species may be tracking environmental conditions over time, which is consistent with species sorting [1]. We found less temporal turnover in larger water volume treeholes, which are known to be more stable habitats [11], and this would be expected to lead to fewer changes in community composition, as we found.

Prey populations were also affected by predator presence. The larvae of *T. rutilus *are generalist, size-selective predators, and exert strong effects on prey populations [37-39]. We found that *A. triseriatus *and *C. guttipennis *densities were negatively correlated with densities of *T. rutilus*. Treeholes with *T. rutilus *present also had almost two more species, on average, than treeholes without *T. rutilus*. Predation may thus work as a species sorting mechanism. The wide habitat preference of *A. triseriatus *and the hatching of multiple cohorts over time are mechanisms of coexistence with *T. rutilus *in treehole metacommunities, as is the observation that these two species often occupy treeholes based on their permanence [37]. *Culicoides guttipennis *has even wider habitat preferences, and can survive in treeholes with little or no standing water [16], also permitting regional coexistence. The influence of *T. rutilus *is seasonal, as its populations are dependent upon water volume and prey availability, and larvae are not consistently present in any one treehole [37,38]. The gap in the presence of this top predator in the spring may be caused by adult emergence of overwintering fourth instar larvae. Community composition is thus influenced by a combination of direct and indirect effects of abiotic factors and predation. Prey populations can survive in colonized patches without predators, in a source-sink dynamic [2], which in our system suggests the importance of local factors combined with a moderate amount of dispersal.

The dependence of every species examined on some environmental factor strongly supports the hypothesis that local factors outweigh regional factors. Some species were more sensitive to water volume, leaf mass, or presence of the top predator, which further supports species sorting. This is consistent with ideas that life history characteristics and adaptations differ among component species, and each species will more likely be found in its preferential environment [4]. Even if dispersal rates were high or dispersal was not impeded by distance between patches, environmental variation may still affect the composition of the community. Under a global dispersal scenario, we would expect to observe populations of certain species in sink treeholes, due to high colonization in all treeholes regardless of local environmental conditions [2]. We do observe this in mosquito larvae, which were found in source patches with large volumes of water and in sink patches with little or no water. These larval populations are subsequently sorted by local environmental conditions, leading to populations remaining only in habitat patches with conditions favorable for survival, and being eliminated from treeholes with little or no standing water. If the dynamics of these populations within local habitat patches occur on a more rapid temporal scale than colonization events, then local communities should change rapidly over time, exhibit high spatial turnover, and have little synchrony of population densities, as our data do.

### Spatial effects

As adult insects emerge from treeholes, they must find mates, possibly a vertebrate host for a blood meal, and new treeholes. Little is known of dispersal abilities of treehole insects, especially non-culicids [3,40,41]. There appears to be some oviposition selection based on environmental factors, at least for some species [8,37,42,43], indicating that species are adapted to particular conditions within treeholes. Interactions such as competition or predation may be selective factors that shaped oviposition preferences [44]. The lack of synchrony in both densities and presence-absence of individual species, as we predicted, is in agreement with Ellis et al. [3] and suggests a strong influence of local conditions over effects of distance between patches, especially if dispersal is limited. In addition, the specific conditions of individual treeholes, including shape, size of the opening, location in the forest, height from the ground, and total size, all contribute to reducing synchrony of populations among treehole pairs [29]. High levels of spatial turnover would be predicted for communities that have limited dispersal, and the relationship between spatial turnover and distance indicates that communities farther apart are less similar, again, as we predicted. However, the high spatial turnover even at short distances supports the notion that patches are dissimilar and highly variable spatially, and suggests that dispersal is not high enough to homogenize local communities.

The most common species, *A. triseriatus*, had the highest proportion occupancy and colonization, and species with low proportion occupancy also had low proportion colonization. Mosquito (*A. triseriatus *and *T. rutilus*) occupancy responded most strongly to water volume, consistent with Ellis et al. [3] and our predictions. While occupancy-water volume slopes for *C. guttipennis *and *H. pulchella *were significantly greater than zero, they were lower than the slopes for mosquitoes, which agrees with our prediction that these species would respond less strongly to changes in water volume. Further, Y-intercepts for the mosquitoes were not different from zero, indicating close to zero occupancy in treeholes with no standing water. The probability of occupancy for *C. guttipennis *and *H. pulchella *is significantly greater than zero in treeholes with no standing water. This suggests a possible trade-off as non-mosquitoes have non-zero occupancy in nearly dry treeholes, allowing them to survive where two other species, one the numerically dominant species, and the other the top predator, cannot. *Aedes triseriatus *can quickly recolonize these patches when water refills them [3,24], but other species survive in these patches during drought. The quick recolonization by mosquitoes suggests rapid, global dispersal, and specialization in treeholes with different conditions suggests a possible trade-off between ability to survive in a habitat and colonization ability, as well as a mechanism for regional coexistence of species.

## Conclusion

Our study adds to recent examinations of factors that affect composition in container metacommunities [3,7,45]. We found that treehole insect community composition was best explained by local conditions, temporal change, and colonization events. Spatiotemporal variation was evident, and asynchrony, even at short distances, implies that colonization events, although frequent for some species, are not synchronizing communities. Temporal dynamics were consistent with the strong seasonality in community and abiotic conditions observed in temperate forest treeholes, and suggest a strong role of disturbance in these communities. Individual species populations were asynchronous even at short distances, but closer communities were still more similar than communities farther apart. Oviposition preferences or environmental factors that quickly act to eliminate a species from a habitat would lead to the patterns we observed, as suggested by the hypothesis that local conditions prevail over regional factors. We conclude that local effects, temporal dynamics, and species preferences support the view that environmental factors play a dominant role in determining local community composition in this metacommunity.

## Authors' contributions

CJP conceived of the study and drafted the manuscript. All authors were involved in the acquisition of data, analyzing and interpretation of data, revising the manuscript, and all have read and approved the final manuscript.

## Authors' information

JDB is currently in the Department of Ecology and Evolutionary Biology, University of Tennessee, Knoxville, TN. JQB is currently in the Department of Forestry, West Virginia University. JG is currently in the University of Georgia Veterinary School, Athens, GA. LH is currently in the Aquatic Animal Health Program, College of Veterinary Medicine, University of Florida, Gainesville, FL. KH currently resides in Denver, CO, and BK currently resides in Franklin, NC. TK is currently attending Trinity University, San Antonio, TX. LS is currently in the Graduate School of Oceanography, University of Rhode Island, Narragansett, RI. SV is currently with GEI Consultants, Inc., Littleton, CO.
